# Cross-sectional Associations of Fatigue with Cerebral β-Amyloid in Older Adults at Risk of Dementia

**DOI:** 10.3389/fmed.2017.00173

**Published:** 2017-11-06

**Authors:** Claudie Hooper, Philipe De Souto Barreto, Nicola Coley, Matteo Cesari, Pierre Payoux, Anne Sophie Salabert, Sandrine Andrieu, Bruno Vellas, for the MAPT/DSA Study Group

**Affiliations:** ^1^Gérontopôle, Department of Geriatrics, CHU Toulouse, Purpan University Hospital, Toulouse, France; ^2^UMR1027 INSERM, Université de Toulouse III Paul Sabatier, Toulouse, France; ^3^Department of Epidemiology and Public Health, CHU Toulouse, Toulouse, France; ^4^UMR 1214, Toulouse Neuroimaging Center, University of Toulouse III, Toulouse, France; ^5^Department of Nuclear Medicine, CHU Toulouse, Toulouse, France

**Keywords:** fatigue, β-amyloid, Alzheimer’s disease, frailty, cognition

## Abstract

Fatigue is a common symptom in the elderly and has also been associated with impaired cognition in older adults. Hence, we sought to explore the cross-sectional relationship between fatigue and cerebral β-amyloid (Aβ) in 269 elderly individuals reporting subjective memory complaints from the Multidomain Alzheimer Preventive Trial. Standard uptake value ratios (SUVRs) were generated by [^18^F] florbetapir positron emission tomography (PET) using the cerebellum as a reference. Cortical-to-cerebellar SUVRs (cortical-SUVRs) were obtained using the mean signal from the frontal cortex, temporal cortex, parietal cortex, precuneus, anterior cingulate, and posterior cingulate. Other brain regions independently assessed were the anterior cingulate, anterior putamen, caudate, hippocampus, medial orbitofrontal cortex, occipital cortex, parietal cortex, pons, posterior cingulate, posterior putamen, precuneus, semioval center, and temporal cortex. Fatigue was defined according to two questions retrieved from the Center for Epidemiological Studies-Depression scale. Chronic fatigue was defined as meeting fatigue criteria at two consecutive clinical visits 6 months apart between study baseline and 1 year (visits were performed at baseline, 6 months and 1 year then annually). Cross-sectional associations between fatigue variables and cerebral Aβ were explored using fully adjusted multiple linear regression models. We found no statistically significant cross-sectional associations between fatigue assessed at the clinical visit closest to PET and Aβ in any brain region. Similarly, chronic fatigue was not significantly associated with Aβ load. Sensitivity analysis in subjects with a Clinical Dementia Rating of 0.5 showed that fatigue reported at the clinical visit closest to PET was, however, weakly associated with increased Aβ in the hippocampus (B-coefficient: 0.07, 95% CI: 0.01, 0.12, *p* = 0.016). These preliminary results suggest that fatigue might be associated with Aβ in brain regions associated with Alzheimer’s disease in subjects in the early stages of disease.

## Introduction

Fatigue is the sense of persistent general tiredness and exerts a significant negative impact on health status (1). It is a common symptom in older adults and has been suggested to serve as a clinically relevant biomarker for pathological aging (2). Fatigue is specifically associated with physical frailty (2, 3) and cognitive frailty, where physical frailty presents with cognitive impairments (4, 5). Research into fatigue and Alzheimer’s disease (AD), the most common form of dementia in the elderly (6), is limited. However, fatigue has been cross-sectionally associated with brain atrophy and compromised cognition in cognitively normal older adults (7) and longitudinally with increased risk of cognitive decline in older adults without dementia (8). Fatigue has also been more frequently reported in patients with dementia at 3-year follow-up (9) and fatigue is a symptom of depression, a condition that often co-presents with dementia (10, 11).

In accordance with the amyloid cascade hypothesis of AD, β-amyloid (Aβ) is thought to be the main driver of AD pathology culminating in neurofibrillary tangle formation, which in turn precipitates neuronal loss and cognitive impairments (12, 13). It has been suggested that fatigue might occur as a result of depleted physiological reserves (14), and fatigue has been associated with increased oxidative stress (15). Moreover, frailty is associated with pro-inflammatory changes (16–18), but due to the biological complexity of the frailty phenotype specific associations with its fatigue component are difficult to distinguish. It seems plausible, therefore, that fatigue might lead to alterations in homeostasis leading to secondary increases in Aβ deposition especially considering that oxidative stress and inflammation fuel amyloidogenesis (19, 20). Furthermore, polypharmacy and conditions prevalent in the elderly such as anemia and sleep apnea are associated with fatigue (21–25). Sleep apnea has been associated with increased cerebral Aβ (26) and iron deficiency leads to alterations in the expression of genes involved in amyloidogenesis (27). Moreover, polypharmacy is a risk factor for cognitive impairment (28), which potentially might manifest through Aβ-dependent mechanisms. Hence, in this study, we sought to explore the cross-sectional associations between fatigue and cerebral Aβ in older adults reporting subjective memory complaints. We hypothesized that fatigue would be associated with increased cerebral Aβ load.

## Materials and Methods

### The Multidomain Alzheimer Preventive Trial (MAPT): Standard Protocol Approvals, Registrations, and Ethics

Data were obtained from a [^18^F] florbetapir positron emission tomography (PET) study carried out as part of the Multidomain Alzheimer Preventive Trial (MAPT), which was a large phase III, multicentre, randomized, placebo-controlled trial (29) (registration: NCT00672685). The trial had a four-arm design comprising a placebo group and three treatment groups; omega 3 polyunsaturated fatty acid (n-3 PUFA) supplementation, multidomain intervention (involving nutritional and exercise counseling and cognitive training), and n-3 PUFA supplementation plus multidomain intervention. The trial was designed to assess the efficacy of the interventions in slowing cognitive decline in older adults at risk of dementia (*n* = 1,680) (29). In the main analysis of MAPT, no significant effects of any of the interventions were found on the composite cognitive score compared to placebo after adjustment for multiple testing (30). Both the MAPT and [^18^F] florbetapir PET study were approved by the ethics committee in Toulouse (CPP SOOM II) and written consent was obtained from all participants.

### Participants

A total of 271 subjects participated in the MAPT-[^18^F] florbetapir ancillary study. At inclusion, participants were community-dwelling, men and women without dementia, aged ≥70, and who met at least one of the following criteria: spontaneous memory complaints, limitation in executing ≥1 Instrumental Activity of Daily Living or slow gait speed (<0.8 meters/sec). Two participants were excluded because they developed dementia at the clinical assessment closest to PET [Clinical Dementia Rating (CDR) ≥ 1]. Thus, a total of 269 subjects were included in the analyses described here. The participants of the main MAPT study not assessed for cerebral Aβ load were similar to the participants in the PET sub-study in terms of age at baseline (main MAPT study: 75.9 ± 4.5 years, PET sub-study: 75.2 ± 4.2 years), sex (main MAPT study: 65.6% female, PET sub-study: 60.2% female) and cognition at baseline measured as mini mental state examination test score (main MAPT study: 28.0 ± 1.6, PET sub-study: 28.3 ± 1.5).

### [^18^F] Florbetapir PET

PET-scans were performed once during MAPT in volunteers using [^18^F] florbetapir as previously described (29, 31). All data acquisitions were begun 50 min after injection of a mean of 4 MBq/kg weight of [^18^F]-Florbetapir. Radiochemical purity of [^18^F]-Florbetapir was always superior to 99.5%. Standard uptake value ratios (SUVRs) were generated from semi-automated quantitative analysis using the cerebellum as a reference. Cortical-to-cerebellar SUVRs (cortical-SUVRs) were obtained using the mean signal from the following cortical regions: frontal, temporal, parietal, precuneus, anterior cingulate, and posterior cingulate as previously described (32). Other brain regions independently assessed were the: anterior cingulate, anterior putamen, caudate, hippocampus, medial orbitofrontal cortex, occipital cortex, parietal cortex, pons, posterior cingulate, posterior putamen, precuneus, semioval center, and temporal cortex. A quality control based on semi-quantification process was also performed. The median and interquartile range (IQR) for the time interval between baseline and PET-scan assessment was 487.5 days (IQR: 349–728) and 3.7% (10 out of 269) of subjects received a PET-scan at study baseline.

### Fatigue

Fatigue was defined according to the following two questions retrieved from the Center for Epidemiological Studies-Depression scale: (a) I felt that everything I did was an effort, (b) I could not get going. The question is asked “How often in the last week did you feel this way?” and subjects score their responses: 0 = rarely or none of the time (<1 day), 1 = some or a little of the time (1–2 days), 2 = a moderate amount of the time (3–4 days), 3 = most of the time. Subjects answering 2 or 3 to either of these questions were designated as fatigued otherwise subjects were classed as non-fatigued. Subjects were classed as having chronic fatigue if answering 2 or 3 to either of the questions at two consecutive visits between study baseline and 1 year (visits were performed at baseline, 6 months, and 1 year after which they were performed annually) otherwise subjects were deemed non-chronically fatigued. The adopted definition of fatigue is commonly used to define the “exhaustion” criterion in the field of frailty (3).

### Confounding Variables

On the basis of data availability and the literature on dementia (33), we selected the following confounders: age at PET-scan assessment, gender, educational level, cognitive status assessed at the clinical visit closest to PET-scan (CDR: scores 0 or 0.5), MAPT group allocation (four groups: placebo, multidomain intervention, n-3 PUFA supplementation and multidomain intervention + n-3 PUFA supplementation), depressive symptoms assessed closed to PET-scan (Geriatric Depression Scale: scores 0–30) and Apolipoprotein E ε4 (ApoE ε4) genotype (carriers of at least one ε4 allele versus non-carriers).

### Statistical Analysis

Descriptive statistics are presented as median (IQR) or absolute values/percentages as appropriate. After completing analysis of the primary hypotheses in MAPT (30), we performed *post hoc* analyses using multiple linear regression models to explore the cross-sectional relationships between fatigue and cerebral Aβ load (measured as SUVR). Clinical and demographic characteristics were compared between the participants deemed as non-fatigued or fatigued (with fatigue assessed at the clinical exam closest to PET-scan) using chi squared tests for categorical variables and Wilcoxon rank sum tests for continuous variables. We ran multiple linear regression analysis to explore the cross-sectional relationship between fatigue at the clinical exam closest to PET-scan and cortical-SUVR and region specific SUVR (13 regions described above) adjusting for all confounders. Sensitivity analysis was performed in subjects with a CDR score of 0.5 as this sub-group represents those more likely to develop AD (34). We also ran multiple linear regression analysis to explore the cross-sectional relationship between chronic fatigue and cortical-SUVR and regional SUVR (13 regions) adjusting for all confounders. There was no correction for multiple comparisons due to the exploratory nature of this study: *p* < 0.05 was considered statistically significant. All analyses were performed using Stata version 14 (Stata Corp., College Station, TX, USA).

## Results

### Sample Characteristics

Clinical and demographic characteristics of the study participants are shown in Table 1. The median age of the participants was approximately 75 years and around 60% of the subjects were female and approximately half of the subjects had a CDR score of 0.5. Participants exhibited a high educational level and approximately 30% of the subjects were ApoE ε4 carriers. There were no statistically significant differences between subjects classed as non-fatigued or fatigued (with fatigue assessed at the clinical exam closest to PET-scan). A total of 42 participants out of 269 (15.6%) were classified as fatigued and of these 42.9% (18 out of 42) were Aβ positive using a threshold of mean cortical-SUVR ≥ 1.17 (31, 35). A total of 26 participants out of 269 (9.7%) were classified as having chronic fatigue and of these 38.5% (10 out of 26) were Aβ positive.

**Table 1:** Participant characteristics.

Variables	Non-fatigued subjects (*n* = 227)	Fatigued subjects (*n* = 42)	*p*-Value
Age, years	75 (72–79)	76 (73–79)	0.434
Sex, women (%)	134 (59.0%)	28 (66.7%)	0.353
Education (%)			0.190
No diploma or primary school certificate	54 (24.1%)	14 (34.1%)	
Secondary education no high-school diploma	72 (32.1%)	7 (17.1%)	
High-school diploma	31 (13.8%)	8 (19.5%)	
Higher diploma	67 (29.9%)	12 (29.3%)	
Group allocation (%)			0.550
Multidomain intervention	57 (28.6%)	11 (26.2%)	
n-3 PUFA supplementation	48 (21.1%)	12 (28.6%)	
Multidomain intervention and n-3 PUFA supplementation	65 (28.6%)	8 (19.0%)	
Placebo	57 (25.1%)	11 (26.2%)	
% of CDR 0.5 (%)	108 (47.6%)	23 (54.7%)	0.392
ApoE ε4 carriers (%)^a^	53 (26.8%)	12 (32.4%)	0.480
Cortical-SUVR	1.1 (1.0–1.3)	1.1 (1.1–1.3)	0.927

*Age and CDR score closest to PET-scan are presented. Data are expressed as median (interquartile range) or as absolute values/percentages. Clinical and demographic characteristics were compared between the participants deemed as non-fatigued or fatigued (with fatigue assessed at the clinical exam closest to PET-scan) using chi squared tests for categorical variables and Wilcoxon rank sum tests for continuous variables*.

*ApoE, apolipoprotein E; CDR, clinical dementia rating; n-3 PUFA, omega 3 polyunsaturated fatty acid; SUVR, standard uptake ratio values*.

*^a^ApoE ε4 status available for n = 235*.

### Exploration of the Relationship between Fatigue and Cerebral Aβ

There were no statistically significant cross-sectional associations of fatigue at the clinical exam closest to PET-scan with cortical or region specific Aβ load after adjustment for all confounders (Table 2). Sensitivity analysis performed in subjects with a CDR score of 0.5, however, showed a weak positive association between fatigue reported at the clinical exam closest to PET-scan and Aβ load in the hippocampus (Table 3). Chronic fatigue was not significantly associated with cortical Aβ or Aβ found in any other brain region after adjustment for all confounders (Table 4).

**Table 2:** Multiple linear regressions examining the cross-sectional associations between fatigue at the clinical exam closest to PET-scan and cerebral β-amyloid load.

β-amyloid load	Unadjusted model (*n* = 269)			Adjusted model (*n* = 231)		
	B-coeff.	95% CI	*p*-Value	B-coeff.	95% CI	*p*-Value
Cortical-SUVR	−0.01	−0.06, 0.05	0.800	−0.04	−0.10, 0.02	0.184
SUVR by brain region;						
Anterior cingulate	−0.01	−0.08, 0.06	0.832	−0.06	−0.14, 0.02	0.140
Anterior putamen	−0.02	−0.12, 0.08	0.683	−0.05	−0.17, 0.06	0.367
Caudate	0.05	−0.06, 0.16	0.344	0.04	−0.09, 0.17	0.532
Hippocampus	0.02	−0.02, 0.06	0.263	0.04	−0.00, 0.08	0.071
Medial orbitofrontal cortex	−0.00	−0.05, 0.04	0.939	−0.02	−0.07, 0.03	0.494
Occipital cortex	−0.02	−0.08, 0.04	0.553	−0.06	−0.13, 0.01	0.073
Parietal cortex	−0.03	−0.09, 0.03	0.339	−0.06	−0.13, 0.01	0.080
Pons	−0.01	−0.05, 0.04	0.702	−0.00	−0.06, 0.05	0.896
Posterior cingulate	0.00	−0.06, 0.06	0.975	−0.03	−0.10, 0.04	0.362
Posterior putamen	−0.01	−0.09, 0.07	0.727	−0.02	−0.11, 0.07	0.667
Precuneus	−0.00	−0.08, 0.08	0.964	−0.05	−0.13, 0.03	0.245
Semioval center	0.02	−0.03, 0.08	0.424	0.02	−0.04, 0.09	0.507
Temporal cortex	−0.00	−0.06, 0.05	0.891	−0.03	−0.09, 0.02	0.253

*The adjusted model contained fewer subjects due to missing data on confounders*.

*B-coeff, B-coefficient; CI, confidence intervals; p, probability; SUVR, standard uptake ratio values*.

**Table 3:** Multiple linear regressions examining the cross-sectional associations between fatigue at the clinical exam closest to PET-scan and cerebral β-amyloid load in subjects with a CDR score of 0.5.

β-amyloid load	Unadjusted model (*n* = 131)			Adjusted model (*n* = 113)		
	B-coeff.	95% CI	*p*-Value	B-coeff.	95% CI	*p*-Value
Cortical-SUVR	−0.01	−0.09, 0.07	0.829	−0.03	−0.12, 0.05	0.438
SUVR by brain region;						
Anterior cingulate	0.01	−0.10, 0.11	0.922	−0.03	−0.15, 0.09	0.597
Anterior putamen	−0.01	−0.15, 0.13	0.869	−0.05	−0.21, 0.12	0.585
Caudate	0.08	−0.07, 0.24	0.276	0.11	−0.06, 0.29	0.204
Hippocampus	0.04	−0.01, 0.09	0.106	0.07	0.01, 0.12	0.016
Medial orbitofrontal cortex	0.00	−0.06, 0.06	0.974	0.00	−0.06, 0.06	0.973
Occipital cortex	−0.01	−0.10, 0.08	0.750	−0.05	−0.15, 0.04	0.265
Parietal cortex	−0.05	−0.14, 0.03	0.241	−0.08	−0.16, 0.01	0.085
Pons	0.01	−0.06, 0.07	0.802	0.01	−0.06, 0.08	0.762
Posterior cingulate	−0.01	−0.09, 0.08	0.824	−0.04	−0.13, 0.05	0.426
Posterior putamen	−0.01	−0.11, 0.09	0.810	−0.02	−0.14, 0.09	0.693
Precuneus	−0.00	−0.12, 0.11	0.958	−0.04	−0.17, 0.08	0.496
Semioval center	0.04	−0.03, 0.12	0.249	0.06	−0.02, 0.15	0.147
Temporal cortex	0.00	−0.07, 0.08	0.904	−0.02	−0.10, 0.06	0.688

*The adjusted model contained fewer subjects due to missing data on confounders*.

*B-coeff, B-coefficient; CI, confidence intervals; p, probability; SUVR, standard uptake ratio values*.

**Table 4:** Multiple linear regressions examining the cross-sectional associations between chronic fatigue and cerebral β-amyloid load.

β-amyloid load	Unadjusted model (*n* = 269)			Adjusted model (*n* = 231)		
	B-coeff.	95% CI	*p*-Value	B-coeff.	95% CI	*p*-Value
Cortical-SUVR	−0.01	−0.08, 0.06	0.854	−0.03	−0.10, 0.05	0.452
SUVR by brain region;						
Anterior cingulate	−0.01	−0.10, 0.07	0.760	−0.05	−0.14, 0.04	0.293
Anterior putamen	0.02	−0.10, 0.15	0.736	0.01	−0.13, 0.14	0.922
Caudate	0.02	−0.11, 0.16	0.714	0.02	−0.13, 0.17	0.798
Hippocampus	−0.01	−0.06, 0.03	0.518	−0.01	−0.06, 0.04	0.677
Medial orbitofrontal cortex	−0.01	−0.07, 0.04	0.687	−0.02	−0.08, 0.04	0.433
Occipital cortex	−0.01	−0.09, 0.06	0.756	−0.04	−0.12, 0.04	0.286
Parietal cortex	0.00	−0.07, 0.08	0.994	−0.00	−0.08, 0.07	0.903
Pons	−0.02	−0.08, 0.03	0.425	−0.02	−0.08, 0.04	0.464
Posterior cingulate	−0.01	−0.09, 0.06	0.716	−0.04	−0.12, 0.04	0.384
Posterior putamen	−0.00	−0.10, 0.10	0.967	0.01	−0.10, 0.12	0.920
Precuneus	0.01	−0.09, 0.10	0.857	−0.02	−0.12, 0.08	0.639
Semioval center	−0.00	−0.07, 0.07	0.943	−0.01	−0.09, 0.07	0.847
Temporal cortex	−0.01	−0.07, 0.06	0.773	−0.03	−0.10, 0.04	0.379

*The adjusted model contained fewer subjects due to missing data on confounders*.

*B-coeff, B-coefficient; CI, confidence intervals; p, probability; SUVR, standard uptake ratio values*.

## Discussion

In this study, we did not find any significant cross-sectional associations between fatigue (assessed closest to PET-scan examination or chronic) and cortical or region specific Aβ load in our total study population. However, sensitivity analysis in subjects with a CDR of 0.5 showed that fatigue reported closest to PET-scan was associated with increased Aβ load specifically in the hippocampus.

Why fatigue might be specifically associated with hippocampal Aβ pathology in subjects at increased risk of AD (CDR = 0.5) requires further research attention. However, there is evidence that fatigue, modeled in rats through the induction of sleep deprivation, reduces hippocampal as well as cortical dendritic spines (36) and inhibits long-term potentiation and hippocampal dependent learning tasks (37). Thus, it might be that fatigue also modulates cell signaling cascades to promote amyloidogenesis specifically in the hippocampus. In line with this, in mouse models of AD, acute sleep deprivation is associated with increased levels of interstitial brain levels of Aβ, whereas chronic sleep deprivation has been associated with increased Aβ plaques (38). Increased brain Aβ load has also been cross-sectionally associated with poor sleep (39) and longer sleep latency (time taken to fall asleep) (40) in human subjects. Oxidative stress is associated with fatigue (15) and pro-inflammatory mediators such as interleukin 6 and C-reactive protein have been associated with frailty (which includes fatigue in the phenotype) (16, 18); therefore such signalling intermediates might promote fatigue-induced amyloidogenesis at the molecular level (19, 20). Interestingly, fatigue has also been associated with compromised cognition in older adults without dementia (7, 8). With this in mind, fatigue might modulate cognition *via* Aβ-dependent mechanisms in human subjects with early AD. More research is needed to verify the links between fatigue and Aβ deposition, particularly to rule out the possibility that increased Aβ in the brain might precipitate fatigue. A better understanding of the biological basis of fatigue would facilitate such studies.

The strengths of the current study are the large sample size with PET [^18^F] florbetapir imaging data and the simultaneous availability of successive fatigue measurements over time enabling the creation of a chronic fatigue variable. Nevertheless, there are some limitations. The main limitation of this study is that it is a secondary analysis of the MAPT imaging sub-study; thus, the study was not specifically powered to address our hypothesis on the association of fatigue with increased cerebral Aβ load. The cross-sectional nature (due to lack of longitudinal imaging data) precluded the examination of the relationship between fatigue and Aβ temporally. Furthermore, it should be noted that PET-scans were performed throughout the three year period of MAPT, so the study was not of a true cross-sectional nature. Moreover, although used by others (2, 41) as a measure of fatigue, the self-reported fatigue variable used here was derived from a questionnaire designed to measure depression and as such may not robustly capture the physiological component of fatigue but rather focus on the psychological element. There was also no data available on other diseases that might contribute to fatigue such as anemia, sleep apnea, or cancer.

In conclusion, we have shown here that fatigue might be associated with increased Aβ in the hippocampus specifically in subjects with an augmented risk of AD. Further research is required to confirm our preliminary findings. A longitudinal study examining the temporal association between fatigue and Aβ would provide more evidence as to whether fatigue might modulate cerebral Aβ levels.

## MAPT/DSA Group Refers To

### MAPT Study Group

#### Principal Investigator

Bruno Vellas (Toulouse); *Coordination*: Sophie Guyonnet; *Project leader*: Isabelle Carrié; *CRA*: Lauréane Brigitte; *Investigators*: Catherine Faisant, Françoise Lala, Julien Delrieu, Hélène Villars; *Psychologists*: Emeline Combrouze, Carole Badufle, Audrey Zueras; *Methodology, statistical analysis and data management*: Sandrine Andrieu, Christelle Cantet, Christophe Morin; *Multidomain group*: Gabor Abellan Van Kan, Charlotte Dupuy, Yves Rolland (physical and nutritional components), Céline Caillaud, Pierre-Jean Ousset (cognitive component), Françoise Lala (preventive consultation), Bertrand Fougère (Toulouse). The cognitive component was designed in collaboration with Sherry Willis from the University of Seattle, and Sylvie Belleville, Brigitte Gilbert and Francine Fontaine from the University of Montreal.

#### Coinvestigators in Associated Centers

Jean-François Dartigues, Isabelle Marcet, Fleur Delva, Alexandra Foubert, Sandrine Cerda (Bordeaux); Marie-Noëlle-Cuffi, Corinne Costes (Castres); Olivier Rouaud, Patrick Manckoundia, Valérie Quipourt, Sophie Marilier, Evelyne Franon (Dijon); Lawrence Bories, Marie-Laure Pader, Marie-France Basset, Bruno Lapoujade, Valérie Faure, Michael Li Yung Tong, Christine Malick-Loiseau, Evelyne Cazaban-Campistron (Foix); Françoise Desclaux, Colette Blatge (Lavaur); Thierry Dantoine, Cécile Laubarie-Mouret, Isabelle Saulnier, Jean-Pierre Clément, Marie-Agnès Picat, Laurence Bernard-Bourzeix, Stéphanie Willebois, Iléana Désormais, Noëlle Cardinaud (Limoges); Marc Bonnefoy, Pierre Livet, Pascale Rebaudet, Claire Gédéon, Catherine Burdet, Flavien Terracol (Lyon), Alain Pesce, Stéphanie Roth, Sylvie Chaillou, Sandrine Louchart (Monaco); Kristelle Sudres, Nicolas Lebrun, Nadège Barro-Belaygues (Montauban); Jacques Touchon, Karim Bennys, Audrey Gabelle, Aurélia Romano, Lynda Touati, Cécilia Marelli, Cécile Pays (Montpellier); Philippe Robert, Franck Le Duff, Claire Gervais, Sébastien Gonfrier (Nice); Yannick Gasnier and Serge Bordes, Danièle Begorre, Christian Carpuat, Khaled Khales, Jean-François Lefebvre, Samira Misbah El Idrissi, Pierre Skolil, Jean-Pierre Salles (Tarbes).

#### MRI Group

Carole Dufouil (Bordeaux), Stéphane Lehéricy, Marie Chupin, Jean-François Mangin, Ali Bouhayia (Paris); Michèle Allard (Bordeaux); Frédéric Ricolfi (Dijon); Dominique Dubois (Foix); Marie Paule Bonceour Martel (Limoges); François Cotton (Lyon); Alain Bonafé (Montpellier); Stéphane Chanalet (Nice); Françoise Hugon (Tarbes); Fabrice Bonneville, Christophe Cognard, François Chollet (Toulouse).

#### PET-Scans Group

Pierre Payoux, Thierry Voisin, Julien Delrieu, Sophie Peiffer, Anne Hitzel, (Toulouse); Michèle Allard (Bordeaux); Michel Zanca (Montpellier); Jacques Monteil (Limoges); Jacques Darcourt (Nice).

#### Medico-Economics Group

Laurent Molinier, Hélène Derumeaux, Nadège Costa (Toulouse).

#### Biological Sample Collection

Christian Vincent, Bertrand Perret, Claire Vinel (Toulouse).

#### Safety Management

Pascale Olivier-Abbal.

### DSA Group

Sandrine Andrieu, Christelle Cantet, Nicola Coley.

## Ethics Statement

This study was carried out in accordance with the recommendations of the Toulouse ethics commitee (CPP SOOM II) with written informed consent from all subjects. All subjects gave written informed consent in accordance with the Declaration of Helsinki.

## Author Contributions

CH was responsible for data analysis and writing the manuscript. PB supervised the data analysis and was involved in the critical appraisal of the manuscript. NC was involved in data analysis and critical appraisal of the manuscript. PP and AS performed the [^18^F] florbetapir PET. SA, MC, and BV were involved in study design and critical appraisal of the manuscript.

## Conflict of Interest Statement

The authors declare that the research was conducted in the absence of any commercial or financial relationships that could be construed as a potential conflict of interest.
